# Prevalence and associated factors of internet addiction among Chinese adolescents: association with childhood trauma

**DOI:** 10.3389/fpubh.2023.1172109

**Published:** 2023-08-17

**Authors:** Tianqing Fan, Mireille Twayigira, Lintong Song, Xuerong Luo, Chunxiang Huang, Xueping Gao, Yanmei Shen

**Affiliations:** ^1^Department of Psychiatry, National Clinical Research Center for Mental Disorders, and National Center for Mental Disorders, The Second Xiangya Hospital of Central South University, Changsha, Hunan, China; ^2^Autism Center of the Second Xiangya Hospital, Central South University, Changsha, China

**Keywords:** internet addiction, childhood trauma, adolescents, suicidal behaviors, cross-sectional study

## Abstract

**Introduction:**

Internet addiction (IA) is common among adolescents and may have severe consequences. This study aimed to investigate the prevalence and factors associated with IA among middle school students of Hunan Province, China. Relevance between IA and childhood trauma was also explored.

**Methods:**

One thousand six hundred ten students were enrolled in this cross-sectional study. Data collected included demographics; internet addiction (revised-Chen internet addiction scale); childhood trauma (CTQ-SF); depression, anxiety, and stress symptoms (DASS-21); suicidal behaviors, as well as non-suicidal self-injury (NSSI). Cramer’s V analysis, univariable logistic regression and multivariable logistic regression were used for associations and identifying independent relevance of IA, respectively.

**Results:**

The prevalence of IA was 12.8%. Cramer’s V analysis showed that IA was associated with emotional abuse, emotional and physical neglect, NSSI, suicidal behaviors, stress, anxiety and depressive symptoms, physical disorder history. Regression analysis showed that IA was independently associated with emotional neglect (OR = 3.062, 95% CI: 2.083, 4.501, *p* < 0.001); physical neglect (OR = 2.328; 95% CI: 1.590, 3.409, *p* < 0.001); depressive symptoms (OR = 2.218, 95% CI: 1.467, 3.353, *p* < 0.001) nationality (OR = 1.888, 95% CI: 1.034, 3.447, *p* = 0.006) and age (OR = 1.253, 95% CI: 1.066, 1.471, *p* = 0.006).

**Discussion:**

IA is common among middle school students. Attention should be paid to students with childhood trauma since they have a higher risk for IA, which may increase the risk for suicidal behaviors.

## 1. Introduction

Internet addition (IA), which manifests as repeated or excessive use of the internet usually accompanied by a strong and irresistible desire to use the internet frequently, has gained more and more attention for the negative consequences it brings. In order to encourage more research and experience on IA, the American Psychiatric Association and the World Health Organization have emphasized its importance by including internet gaming disorder in section III of the Diagnostic and Statistical Manual of Mental Disorders-5th Edition as well as including Gaming Disorder and the International Classification of Diseases 11th Edition. A growing number of studies favor the view that internet addiction is an umbrella term for various types of internet-based behavioral addiction, and a recent study has suggested a common neurobiological vulnerability across problematic internet use behaviors rather than a diverse neurocognitive profile for internet gaming disorder (1).

Excessive internet use has brought about a series of negative consequences such as behavioral problems and psychiatric disorders (2, 3), pathological social withdrawal (4), involvement in cyberbullying (5), substance use disorders (6, 7). Adolescents are particularly vulnerable to harm from the internet because they lack proper cognition and sufficient control over their behavior (8). According to recent statistics, the number of internet users in China was 989 million by December 2020, accounting for about one fifth of the global internet users, and the group under the age of 20 accounted for about 16.6%, while the internet penetration rate among adolescents reached 93.1% (9). Given the significant negative consequences of excessive internet use and the vulnerability of adolescents to harm from the internet, a better understanding of the risk factors and other clinical relations of internet addiction is needed to enlighten prevention and intervention efforts.

One important risk factors of internet addition might be childhood trauma. Childhood trauma has been defined as the supreme prognosticator of lifetime mental disorders (10, 11), and it has a well-documented impact on development and the eventual functional sequelae across the lifespan (12). Research has shown that a history of childhood traumatic experiences may be as common among people with IA as among people with substance dependence (13). Studies have demonstrated that childhood trauma might have significant impact on adolescents’ internet gaming disorder mediated through anxiety and depression (14). Moreover, a previous study showed that childhood trauma has significant effects on suicidal ideation mediated through internet addiction in Chinese university students (15).

Previous research has also shown that age, sex, insomnia, stress, anxiety, depression, family relationships and suicidal behavior are associated with internet addiction, and suicidal behavior has undoubtedly attracted more attention due to its dire consequences (16). Studies have established that internet addition is associated with increased suicidality (17); for instance, previous study among college students discovered a 21.4% prevalence of suicide attempts among internet addiction participants (18). Depression and anxiety in relation to internet addition has also received significant attention in previous research, showing that depression and anxiety may predict adolescent internet addiction (19).

Although a considerable amount of research has been done on IA, there have been few studies on the degree of association between childhood trauma and IA among adolescents. In addition, the existing research has only considered specific types of abuse, with few studies examining the association with neglect (emotional and physical), and others have only identified emotional and physical abuse to be related with IA (20). Therefore, the purpose of this study was to estimate the prevalence of internet addiction among middle school students in Hunan Province and to explore the factors associated with internet addiction, particularly exploring the relationship between internet addiction and childhood trauma as well as suicidal behaviors. We hypothesized that IA would be associated with the four types of childhood trauma assessed in this study (childhood emotional and physical abuse, childhood emotional and physical neglect), additionally, we expect that IA would be associated with suicidal behaviors, non-suicidal self-injury (NSSI), depressive, stress and anxiety symptoms.

## 2. Methods

### 2.1. Study design and sample

To assess prevalence and factors associated with internet addiction among Chinese adolescents, this study employed an observational cross-sectional design. One thousand seven hundred eighty-five students from a middle school in Changsha, Hunan province, China, were selected to participate in the study with a cluster sampling method. In November 2020, the purpose of the present study was explained to students and their primary caregivers, they were informed of the right to take part in this study or to withdraw at any time, and after signing an electronic informed consent, the students anonymously filled out the self-report questionnaires online via a widely used social media platform (WeChat, Tencent Inc., China) or via a computer. The inclusion criteria included: (1) students aged 12–18. (2) Students who did not have serious physical illness, who were able to read and understand the questions or able to understand the contents of the questionnaire read to them by the parent or researcher and answer accordingly. Exclusion criteria included: (1) students whose parents and themselves refused to sign the informed consent form to participate in the study. (2) Students with any other problems and were therefore unable to complete the questionnaire. The calculation of sample size was performed using software G*Power, with a parameter set as: two tails, odds ratio = 1.5, *α* err prob. = 0.05, 1 − *β* err prob. =0.95, prop discordant pairs = 0.3. The computed result is: 1,090, to compensate for possible dropout, incomplete data, and other research-related problems, we select a middle school which includes over 1,090 students with a cluster sampling method. One hundred seventy-five students were excluded due to not meeting the inclusion criteria or due to incomplete data. Therefore, 1,610 students (mean age 14, 52.6% boys, 47.4% girls) were included in the final analysis. Prior to the commencement of this study, the research proposal was approved by the Ethics Committee of Xiangya Second Hospital, Central South University, Changsha, China, and the ethics approval number was 009, all the participants included in the study and their primary caregivers signed informed consent forms.

### 2.2. Measures

#### 2.2.1. Demographics

Socio-demographic data included gender, age, nationality, physical disorder history, mental disorder history, family history of mental disorders (FHMD).

#### 2.2.2. Internet addiction

The short form 19-item revised Chen internet addiction scale (CIAS-R) (21) was used to measure internet addiction. This is a 19 item self-rating scale, with a four-point Likert’s scale ranging from 1 (“does not match my experience at all”) to 4 (“definitely matches my experience”). The scale has four subscales: (1) “compulsive use and withdrawal symptoms”; (2) “tolerance”; (3) “interpersonal and health-related problems”; and (4) “time management problems.” The higher the total score, the more severe the internet addiction. Participants with a CIAS-R score of 46 or more were classified as the internet addiction group (IA group), and participants with a CIAS-R score less than 46 was divided in to the control group (Controls). This scale is commonly used to assess internet addiction among Chinese populations, and has shown good psychometric properties in college students (22), as well as among adolescents in china, with an Cronbach’s alpha of the total score of 0.97 among Chinese adolescents in a previous study (23).

#### 2.2.3. Childhood trauma

We utilized a Chinese version of childhood trauma questionnaire-short form (CTQ-SF), a 28-item retrospective self-report scale which is developed by Bernstein in 2003 and translated into Chinese by He in 2019 (24, 25), to assess the maltreatment experienced by participants during their childhood. There are 5 subscales for evaluate physical abuse, sexual abuse, emotional abuse, emotional neglect, and physical neglect respectively, using a 5-point Likert’s scale. The higher the scores for both the total score and the individual subscales, the greater the severity of problem is. In our study, sexual abuse subscale was excluded given the sensitivity of these items in this population. In much of Chinese society, sex that involves children and adolescents is sensitive, therefore, it is difficult and often objected to ask children sex related questions and poses the challenge of getting accurate answers from the participants. These cutoff scores were used to indicate the presence of each of the four forms of maltreatment: emotional neglect ≥10, emotional abuse ≥9, physical abuse ≥8, physical neglect ≥8. Participants who scored higher than the cutoff score in at least one of the subscales were categorized as maltreated (26). This scale had been widely used among Chinese children and adolescents, showing good reliability (27).

#### 2.2.4. Depression, anxiety and stress symptoms

Negative emotional symptoms of depression, anxiety and stress were assessed using the Chinese version of the 21-item depression anxiety stress scale (DASS-21) (28, 29). The scale is divided into 3 subscales of depression, anxiety, and stress, with each scale comprising of 7 items. Each of the 21 items is rated on 4-point Likert’s scale, measuring the extent to which the symptom has been experienced in the past week, from 0 (did not apply to me at all) to 3 (applied to me very much, or most of the time). Higher scores indicate more emotional symptoms experienced in the past week. DASS-21 has been widely used to assess negative symptoms, and the Chinese version has shown good reliability across different populations (30).

#### 2.2.5. Suicidal behaviors and NSSI

Lifetime suicide ideation was assessed using the question: “Have you ever seriously thought about taking your own life? (Yes/No)”; one question was used to assess lifetime suicide plan: “Have you ever made a plan about how you would commit suicide? (Yes/No)”; and lifetime suicide attempt was assessed using the question: “Have you ever tried to take your own life? (Yes/No) If the participants answered any of the questions affirmatively, a follow up question about the frequency during the past 12 months was asked, 1 (zero); 2 (once); 2 (twice); 3 (more than twice). These questions have shown good reliability in previous studies (31).

Non suicidal self-Injury was assessed using the question “In the past 12 months, have you intentionally hurt yourself, but not for the purpose of taking your own life?” Several methods of self-injury were listed as examples, such as “hit yourself,” “pulled your own hair,” “banged your head against something hard,” “pinched yourself,” “scratched yourself,” “bit yourself,” burned yourself, “cut yourself or “other methods” (32). Participants provided a yes or no answer, and if affirmatively answered, they were to indicate which methods they had engaged in. This question has shown good reliability in previous research and among Chinese adolescents (33).

### 2.3. Statistical analysis

Chi square test was used to compare group difference for categorical variables, Kolmogorov–Smirnov test was utilized to test the normal distribution and revealed that none of the data conforms to normal distribution, therefore, nonparametric test Mann–whitney *U* test was used for group comparisons of continuous variables. Cramer’s V analysis was performed to examine relevance existing between internet addition and other parameters. Univariable logistic regression and multivariable logistic regression was utilized to examine factors independently associated with internet addiction among children and adolescents in this study. To selected variables of multivariable logistic analysis, we used an online tool to conducted a directed acyclic graph^1^ to filter variable, afterwards, we conducted a univariable regression analysis, incorporating significant variables into multivariate regression analysis. All statistical analysis were done using SPSS (Version 23.0 IBM, Inc., Chicago, IL), and the significance level was set at 0.05, two tailed. To control for confounding variables, all the variables were included in the Univariable and multivariable logistic regression model.

## 3. Results

This study employed Cramer’s V analysis, univariable and multivariable logistic regression to assess the impact of FDMH, mental disorder history, physical disorder history, age, anxiety, depression, gender, nationality and CTQ on the occurrence of IA in the study participants. The Box-Tidwell method was used to test the linearity between continuous independent variables and the logit transformation of the dependent variable. A total of 8 variables were included in the linear test, with a significance level of 0.00625 after Bonferroni correction. The results of the linear test indicated a linear relationship between all continuous independent variables and the logit transformation of the dependent variable. Out of the 1,610 observations, there were 67 studentized residuals exceeding 2.5 times the standard deviation, but they were retained in the analysis. Ultimately, the logistic regression model obtained statistical significance with a chi-square value of 179.662 and a value of p less than 0.001. The model achieved a correct classification rate of 87.0% for the study participants. The sensitivity of the model was 2.9%, specificity was 99.3%, the positive predictive value was 37.5%, and the negative predictive value was 87.5%. Among the included variables, age, ethnicity, depression, and two neglect factors from the CTQ scale demonstrated statistical significance. In adolescents, for every one-year increase in age, the risk of IA increased by 12.5%.

The prevalence of IA was 12.8% (male, 58.3%; female, 41.7%) in this study. Comparing the demographics and clinical characteristics among students with and without IA, the results indicated significant disparities in nationality, age, non-suicidal selinjury (NSSI), suicidal ideation, suicidal plans, suicidal attempts, stress, anxiety, depression, and all dimensions of childhood trauma, with the exception of physical abuse (Table 1). In univariable analysis, participants with IA were more likely to be of a minority nationality (OR = 1.853, 95% CI: 1.063, 3.228, *p* = 0.029), older (OR = 1.236, 95% CI: 1.060, 1.441, *p* = 0.007), and more likely to report higher levels of depressive (OR = 2.899, 95% CI: 2.152, 3.906, *p* < 0.001), and anxiety symptoms (OR = 1.934, 95% CI: 1.430, 2.615, *p* < 0.001) during the past week, and they were more likely to affirm a history of childhood physical abuse (OR = 1.817, 95% CI: 1.131, 2.918, *p* = 0.014), emotional abuse (OR = 2.258, 95% CI: 1.491, 3.420, *p* < 0.001), physical neglect (OR = 4.595, 95% CI: 3.389, 6.230, *p* < 0.001), and emotional neglect (OR = 5.627, 95% CI: 4.135, 7.657, *p* < 0.001). There were no statistically significant differences between participants with IA and controls in the following variables: gender (OR = 0.770, 95% CI: 0.572, 1.035, *p* = 0.083), history of physical disorder (OR = 1.214, 95% CI: 0.857, 1.720, *p* = 0.275) or mental disorder (OR = 1.367, 95% CI: 0.297, 6.282, *p* = 0.688), family history of mental disorder (OR = 0.680, 95% CI: 0.087, 5.340, *p* = 0.714).

**Table 1:** Demographics and clinical characteristics of participants with and without internet addiction.

Variable	IA (*n* = 206)	Controls (*n* = 1,404)	*χ*^2^/*Z*
*Gender*			
Male, *n* (%)	120 (58.3)	727 (51.8)	3.018^¶^
Female, *n* (%)	86 (41.7)	677 (48.2)	
*Nationality*			
Han, *n* (%)	189 (91.7)	1,339 (95.4)	4.878^¶,*^
Other, *n* (%)	17 (8.3)	65 (4.6)	
Age (years), means ± s	13.28 ± 1.057	13.08 ± 0.937	−2.500^§,*^
Physical disorder history, *n* (%)	48 (23.3)	281 (20.0)	1.194^¶^
Mental disorder history, *n* (%)	2 (1.0)	10 (0.7)	0.162^¶^
FHMD^*^, *n* (%)	1 (0.5)	10 (0.7)	0.136^¶^
NSSI, *n* (%)	53 (25.7)	217 (15.5)	13.581^¶,†^
Suicidal ideation, *n* (%)	84 (40.8)	284 (20.2)	43.019^¶,†^
Suicidal plans, *n* (%)	46 (22.3)	131 (9.3)	31.024^¶,†^
Suicide attempts, *n* (%)	33 (16.0)	92 (6.6)	22.481^¶,†^
DASS, means ± s	20.20 ± 16.000	12.92 ± 12.023	−5.982^§,†^
Stress symptoms, means ± s	7.33 ± 5.717	4.76 ± 4.367	−5.989^§,†^
Stress *n* (%)	85 (41.3)	324 (23.1)	31.350^¶,†^
Anxiety, means ± s	6.26 ± 5.330	4.35 ± 4.259	−4.689^§,†^
Anxiety, *n* (%)	130 (63.1)	659 (46.9)	18.795^¶,†^
Depression, means ± s	6.61 ± 5.803	3.80 ± 4.383	−6.795^§,†^
Depression, *n* (%)	118 (57.3)	444 (31.6)	52.047^¶,†^
CTQ, means ± s	42.08 ± 10.529	31.47 ± 10.991	−12.084^§,†^
Emotional abuse, means ± s	8.54 ± 4.338	7.31 ± 3.353	−3.450^§,**^
Emotional abuse, *n* (%)	34 (16.5)	113 (8.0)	15.484^¶,†^
Physical abuse, means ± s	6.43 ± 2.586	6.06 ± 2.263	−1.801^§^
Physical abuse, *n* (%)	24 (11.7)	95 (6.8)	6.26^¶,*^
Emotional neglect, means ± s	16.71 ± 6.696	10.39 ± 5.737	−12.221^§,†^
Emotional neglect, *n* (%)	128 (62.1)	317 (22.6)	140.551^¶,†^
Physical neglect, means ± s	10.39 ± 3.352	7.71 ± 3.040	−10.577^§,†^
Physical neglect, *n* (%)	125 (60.7)	353 (25.1)	108.68^¶,†^

FHMD, family history of mental health. In vertical row of *χ*^2^/*Z*, ^§^represents the result of Mann–Whitney *U* test, ^¶^means conducted chi square; ^*^p < 0.05, ^**^p < 0.01, ^†^*p* < 0.001.

**Figure 1:**
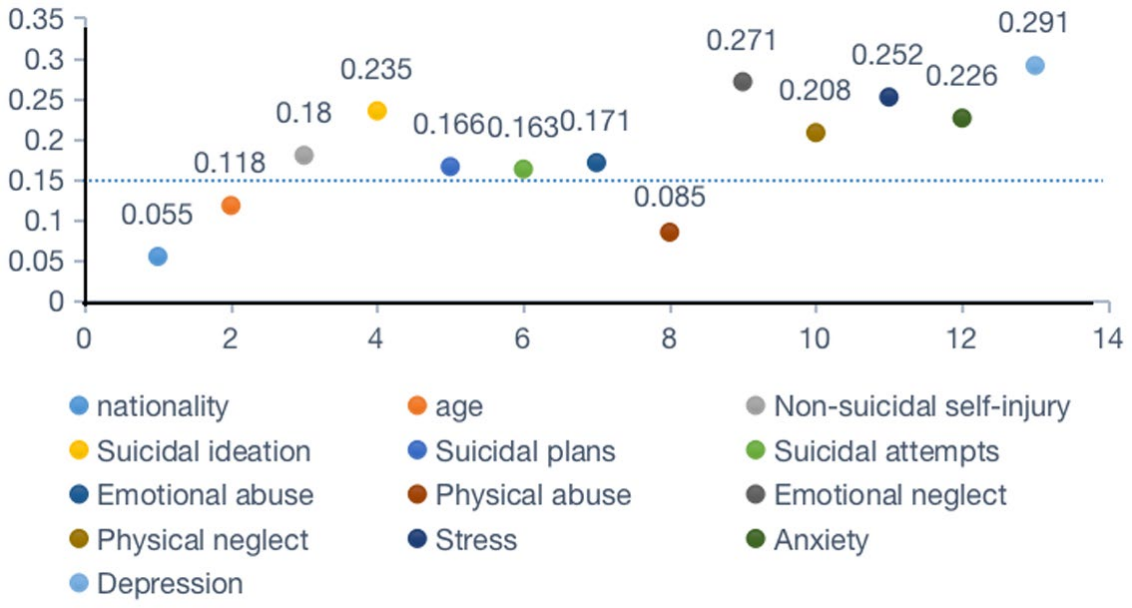
Cramer’s V analysis between IA and other variables. The dashed line is used to distinguish the degree of association, with significant values above the dashed line and insignificant values below it.

Further, for variables that have passed the chi square test, Cramer’s V analysis indicated significant correlations existing between internet addiction and the following parameters: NSSI (Cramer’s V = 0.18), suicidal ideation (Cramer’s V = 0.235), suicidal plans (Cramer’s V = 0.166), suicidal attempts (Cramer’s V = 0.163), emotional abuse (Cramer’s V = 0.171), emotional neglect (Cramer’s V = 0.271), physical neglect (Cramer’s V = 0.208), stress (Cramer’s V = 0.252), anxiety (Cramer’s V = 0.226), depression (Cramer’s V = 0.291), and and surprisingly, correlation between IA and physical abuse (Cramer’s V = 0.085), age (Cramer’s V = 0.118), nationality (Cramer’s V = 0.055) is weak (Figure 1).

Finally, after controlling for confounding factors, the following variables remained independently associated with IA in ultivariable logistic regression analysis: emotional neglect (OR = 3.062, 95% CI: 2.083, 4.501, *p* < 0.001); physical neglect (OR = 2.328; 95% CI: 1.590, 3.409, *p* < 0.001); depressive symptoms (OR = 2.218, 95% CI: 1.467, 3.353, *p* < 0.001) nationality (OR = 1.888, 95% CI: 1.034, 3.447, *p* = 0.006) and age (OR = 1.253, 95% CI: 1.066, 1.471, *p* = 0.006) (Table 2).

**Table 2:** Multivariable logistic regression analysis of IA and clinical characteristics.

Variable	*B*	S.E.	Wald	OR (95% CI)	*p*
Age	0.225	0.082	7.528	1.253 (1.066, 1.471)^**^	0.06
*Nationality*					
Han	Reference				
Other	0.635	0.307	4.280	1.888 (1.034, 3.447)^*^	0.039
*Emotional abuse*					
No	Reference				
Yes	0.011	0.266	0.002	1.011 (0.600, 1.704)	0.968
*Physical abuse*					
No	Reference				
Yes	−0.353	0.297	1.413	0.702 (0.392, 1.258)	0.235
*Emotional neglect*					
No	Reference				
Yes	1.119	0.197	32.399	3.062 (2.083, 4.501)^†^	0.000
*Physical neglect*					
No	Reference				
Yes	0.845	0.195	18.878	2.328 (1.590, 3.409)^†^	0.000
*Anxiety*					
No	Reference				
Yes	0.119	0.211	0.308	1.124 (0.744, 1.699)	0.579
*Depression*					
No	Reference				
Yes	0.797	0.211	14.272	2.218 (1.467, 3.353)^†^	0.000

Only significant variables in univariable regression analysis were included in multivariable regression; ^*^p < 0.05, ^**^p < 0.01, ^†^*p* < 0.001. For multivariable logistic regression model, the constant is −1.919 (*p* < 0.001), *R*^2^ is 179.662, Hosmer and Lemeshow test is *p* = 0.462, omnibus test is *p* < 0.001.

## 4. Discussion

In summary, the prevalence of IA among Hunan middle school students in the current study was 12.8% (male, 58.3%; female, 41.7%). Age, childhood emotional neglect, childhood physical neglect and depressive symptoms were independently associated with IA. The study also found that there were significant associations existing between IA and childhood trauma (except for physical abuse), anxiety symptoms, depressive symptoms, stress symptoms, NSSI, suicidal ideation, suicidal plans and suicidal attempts.

The prevalence of IA differs across different countries, a meta-analysis involving 113 epidemiologic studies and covering 693,306 participants from 31 nations showed an IA prevalence of 7.02%, among them, the prevalence rate varies significantly, ranging from as low as 1% to as high as 38.2% across different countries (34). Nevertheless, it is essential to exercise caution when making direct comparisons of these findings, given the variations in independent standards and sampling methodologies. A comprehensive meta-analysis conducted on 122,454 Chinese college students revealed an overall prevalence of 11.3% for IA, which the numerical value are slightly lower when compared to the results of our research, with the specific rates of 9.3%, 11.2% and 14% when employing different assessment tools such as the 10-item modified Young diagnostic questionnaire, the 20-item internet addiction test and the 26-item Chen internet addiction scale, respectively (35). In addition, there are no uniform diagnostic criteria for IA despite its high recognition in DSM-V; therefore, variations in the diagnostic criteria could be another possible origin of differences in the prevalence since gold standard criteria are unavailable (36). Moreover, being male is a significant predictors of IA among Chinese adolescents (37). Lastly, the growing number of computer users resulting from the advancement of science and technology may also contribute to the higher prevalence rates observed in more recent studies (38). It is crucial and urgent for researchers and clinical doctors to apply common concepts when conducting IA research and to develop gold-standard measurement tools.

This study also conducted analysis on relevance between IA and childhood trauma, and to the best of our knowledge, this is the first study to include this analysis. The results revealed a association between IA and the four types of childhood trauma except for physical abuse included in the study, and the relevance was especially stronger for neglect. Based on previous research, experiencing childhood trauma is not only associated with a variety of mental health problems such as conduct disorders, alcohol dependence, self-harm, suicide attempt (39), depressive symptoms (40), psychosis (41), but also with IA. A previous study on internet gaming disorder revealed that childhood trauma is associated with adolescents’ internet gaming disorder through anxiety and depression (42), and similarly, another study showed an indirect relationship between children’s emotional trauma and internet gaming disorder through depressive symptoms (43). Moreover, as previously highlighted that there is a significant association of childhood trauma and the risk of suicidal ideation and attempted suicide (44), studies have shown that IA have a striking mediating effect on in this association (15). The Cramer’s V analysis also revealed a relevance between IA and NSSI, suicidal ideation, suicidal plan, suicide attempt, depressive symptoms and anxiety symptoms. These findings align with prior research. A study in Chinese adolescences indicate a correlation between IA and NSSI, suggest that this association was similar among both male and female adolescents (45). The literature has also described the relationship between IA and suicidal behavior. A study has specifically indicated that the relationship between internet use and self-harm/suicidal behavior is particularly connect to IA (46). Another study proposed that insomnia and emotional problems may serve as potential explanations for the association between IA and suicidal behavior (47). The distinctive neuro-developmental plasticity experienced during adolescence renders young individuals susceptible to the harmful impacts of internet addiction (IA) on their mental well-being. Conditions like bipolar disorder, affective disorders, social anxiety disorders, major depression, and other related mental health disorders have been found to be associated with IA (8, 48).

As for the factors independently associated with IA, there were some differences in the findings of the current study from previous studies with regards to childhood trauma. The results indicate that both childhood emotional neglect and childhood physical neglect were independently associated with IA. This finding differs from several previous studies, which emphasize the importance of abuse in predicting IA. Possible reasons are as follows: Firstly, the different nationalities, ages, cultural and education background of the participants may be one possible explanation. A study conducted among Turkish college students suggested that among all the types of childhood trauma, emotional abuse seems to be the main predictor of risk severity of IA (20). Secondly, geographical, and economic factors could influence variations in childhood maltreatment estimates around the world and hence affect the detection of an association. For example, in a 2016 study, countries such as Netherlands, United Kingdom presented the lowest maltreatment estimates, and further, high-income countries presented lower physical neglect estimates in comparison to low-or middle-income countries (49). A recent study among Chinese adolescents also found that emotional neglect and physical neglect were more most common among Chinese adolescents, similar to the current study (50). Finally, the variations among these findings could be due to different methodologies or scale selection. For example, one study conducted in Chinese middle school students which used Young’s internet addiction scale, while the current study used the revised Chen internet addiction scale, showed that physical abuse was a potential risk factor (51). In addition to childhood trauma, our study also confirmed that depression, age, and nationality are independently associated with IA, consistent with previous studies.

Certainly, there are some limitations in this study. Firstly, since this study had a cross-sectional design, it is crucial to acknowledge that causal relationships could not be established. To delve deeper into causality, it is imperative to conduct follow-up studies using a longitudinal design. Moreover, specifically examine of potential mediating or moderating effects of IA between childhood trauma and suicidal ideation, depressive symptoms should be conduct in future studies. By doing so, we can gain a more comprehensive understanding of the underlying mechanisms involved. Secondly, data was collected using self-report measures; therefore, recall bias could produce potential estimation errors. Thirdly, the sample in the current study was from one middle school; therefore, the findings might not be generalizable to all adolescent in Hunan, China. Fourthly, this study utilized a cluster sampling method and enrolled all the students from one middle school, future studies should employ more schools and higher standards of epidemiological investigation methods to improve the representativeness. Despite these limitations, this is the first study to directly evaluate the correlation between IA and childhood trauma.

It can be concluded that IA is rifeness among adolescents in Hunan province, china. In this study, students who were older, of a minority nationality, or with a history of childhood emotional neglect, childhood physical neglect, suicidal ideation and depression were more likely to be addicted to the internet. The results also revealed a correlation between IA and childhood trauma, as well as a correlation with suicidality, NSSI, depression, anxiety. Adolescents with a history of childhood trauma may have an increased risk of mental illnesses, problematic internet use and suicidality. People are strongly influenced by the environment they grow up in; therefore, providing more care, protection, and guidance to children may be the key to improving adolescents’ mental health.

## Data availability statement

The raw data supporting the conclusions of this article will be made available by the authors, without undue reservation.

## Ethics statement

The studies involving humans and human participants were reviewed and approved by Medical Ethics Committee of Xiangya Second Hospital, Central South University. Written informed consent to participate in this study was provided by the participants’ legal guardian/next of kin. The studies were conducted in accordance with the local legislation and institutional requirements. The participants provided their written informed consent to participate in this study.

## Author contributions

YS, XL, XG, and CH were responsible for study design. YS, TF, CH, LS, and MT were responsible for recruiting the participants. LS, MT, and TF were involved in statistical analysis. YS, XG, TF, and MT were involved in manuscript preparation and drafting the paper. MT and TF were involved in editing and revising the manuscript. XL and XG were responsible for the critical revision of the manuscript. All authors have contributed to and have approved the final manuscript.

## Funding

This work was supported by the National Natural Science Foundation of China (Grant No. 82201703) and Scientific Research Launch Project for new employees of the Second Xiangya Hospital of Central South University and STI2030-Major Projects-2021ZD0200700.

## Conflict of interest

The authors declare that the research was conducted in the absence of any commercial or financial relationships that could be construed as a potential conflict of interest.

## Publisher’s note

All claims expressed in this article are solely those of the authors and do not necessarily represent those of their affiliated organizations, or those of the publisher, the editors and the reviewers. Any product that may be evaluated in this article, or claim that may be made by its manufacturer, is not guaranteed or endorsed by the publisher.

## Footnotes

^1^ http://www.dagitty.net/dags.html#
